# Fracture of Thin-Walled Polyoxymethylene Bulk Specimens in Modes I and III

**DOI:** 10.3390/ma13225096

**Published:** 2020-11-12

**Authors:** Peer Schrader, Anja Gosch, Michael Berer, Stephan Marzi

**Affiliations:** 1 Institute for Mechanics and Materials, Technische Hochschule Mittelhessen, 35390 Gießen, Germany; stephan.marzi@me.thm.de; 2 Materials Science and Testing of Polymers, Montanuniversität Leoben, 8700 Leoben, Austria; anja.gosch@unileoben.ac.at; 3 Polymer Competence Center Leoben GmbH, Roseggerstraße 12, 8700 Leoben, Austria; michael.berer@pccl.at

**Keywords:** polyoxymethylene, fracture mechanical testing, polymers, quasi-static loads, experimental procedures, J-integral, tensile and shear dominated fracture

## Abstract

Thin-walled polymeric components are used in many applications. Hence, knowledge about their fracture behavior in bulk is beneficial in practice. Within this study, the double cantilever beam (DCB) and out-of-plane double cantilever beam (ODCB) tests are enhanced to enable the testing of such bulk specimens in mode I and mode III on the basis of the *J*-integral. This paper then presents and discusses the experimental results following the investigation of a semicrystalline polymer (polyoxymethylen) under quasi-static load conditions. From the experiments, fracture energies of similar magnitude in both mode I and mode III were determined. In mode III, pop-in fracture was observed. Furthermore, the fracture surfaces were investigated regarding the mode I and mode III dominant crack growth mechanisms, based on the morphology of the tested material. For specimens tested in mode I, no signs of plastic deformation were observed, and the fracture surface appears flat. In mode III, some samples display a twisted fracture surface (twisting angle close to 45${}^{{}^{\circ}}$), which indicates local mode I crack growth. A transfer of the presented methodology to other (more ductile) polymeric materials is deemed possible without further restrictions. In addition, the presented setup potentially enables an investigation of polymeric bulk specimens in mixed mode I+III.

## 1. Introduction

Polymers are used in a wide range of applications, from daily use (packaging, bottles, etc.) to mechanical components (pipes, rollers, gearwheels, etc.). Unfortunately, compared to other materials such as metals or ceramics, there is still a large variety of open questions that remain to be addressed regarding the design of polymeric assemblies. To guarantee the safety of polymeric components, it is beneficial to possess good knowledge of their fracture mechanical properties. Especially when used as tube materials, information about their behavior as thin-walled components is also necessary, as fracture mechanical tests are generally conducted under plain strain conditions and, hence, greater specimen widths are tested.

Due to its material properties such as high stiffness, dimensional stability and fatigue resistance, polyoxymethylene (POM) is currently used in many technical applications [1,2,3]. While the fracture mechanical properties of POM are already available in mode I and mixed mode I+III under monotonic loading and cyclic fatigue tests [4,5,6,7,8], to the authors’ knowledge, little is known about its fracture mechanical properties in pure mode III. This paper addresses the need for the determination of fracture mechanical characteristics of POM for thin-walled components, so far lacking scientific literature.

Generally, only a few methods are available for determining the fracture energy of polymeric materials in pure mode III, of which many have drawbacks either in terms of experimental effort, costs or unwanted contributions in other modes that cannot be deemed negligible [9]. Furthermore, previous studies and therein proposed experimental setups have exclusively focused on linear elastic fracture mechanics, limiting their field of application to brittle and quasi-brittle materials. Hence, a number of questions regarding the mode III fracture of ductile polymers remain to be addressed.

To determine the fracture mechanical properties of POM and possibly other polymers in both pure modes I and III, we propose experimental setups based on the double cantilever beam (DCB) and out-of-plane double cantilever beam (ODCB) tests evaluated by using the *J*-integral. DCB tests with this method of data reduction have historically mostly been used for the experimental investigation of adhesives or composites, see [10,11,12,13] for example, whereas the ODCB test was exclusively used for the investigation of adhesives thus far [14]. The proposed modifications to the DCB and ODCB tests offer the possibility to determine the fracture energy in pure modes I and III on the basis of the *J*-integral. Hence, non-linear fracture behavior can also be characterized with the herein proposed setups, implying that the presented experiments are also used for materials that are not necessarily brittle or quasi-brittle. Conveniently, for the special case of linear elastic and brittle or quasi-brittle materials, the obtained *J*-integral measurements can be directly converted to approximate the fracture toughness values $K_{Ic}$ and $K_{IIIc}$.

This paper begins by examining the theoretical background of the experimental determination of the *J*-integral in pure modes I and III. We will then describe the modifications made to the DCB and ODCB tests, which enabled the determination of the energy release rate (ERR) of a POM homopolymer (POM-H) in modes I and III. Then, after the determination of the ERR in modes I and III, a comparison with relevant literature is sought to classify the generated results in mode I and III. After an examination of the fracture surfaces and methodological critique, we will then present a summary of the most important results.

## 2. Theoretical Background

The ERR *G* is central to the field of fracture mechanics. It can generally be defined as the decrease in potential energy *W* per increase in fracture surface area, yielding

(1) $$G=-\frac{\Delta W}{b\Delta a}$$

for plane problems. Here, *a* denotes the crack length, and *b* is the out-of-plane thickness of the body. For a crack to propagate, the ERR must equal the critical ERR $G=G_{c}$.

**Figure 1:**
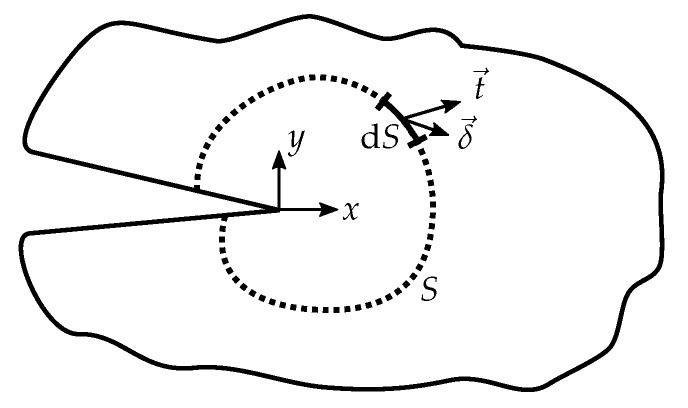
Schematic representation of the line *J*-integral around a notch for a plane problem.

Next to the ERR, the *J*-integral according to Rice [15]

(2) $$J=\int_{S}\left(Wdy-t_{i}\frac{\partial \delta_{i}}{\partial x}dS\right)$$

represents an alternative approach to determine the release of energy during fracture. Here, $t_{i}$ are the components of the traction vector, $\delta_{i}$ are the components of the displacement vector along an arbitrary path *S* containing the crack tip in counter-clockwise direction, and *x* is the direction of crack propagation (see Figure 1 for reference). This two-dimensional integral is path independent. It should be noted that the *J*-integral, according to its initial definition, strictly only applies to materials for which a strain energy density exists, implying that it may only be used for materials that behave in a hyperelastic manner. One should also note that the strain energy density must not explicitly depend on the *x*-coordinate for *J* to be path independent. However, the *J*-integral was also found to be path independent for small scale yielding and even during hardening at the crack tip under a monotonically increasing load [16,17]. An important key factor resulting from this path independence is that contributions from external loads are in equilibrium with the value of *J* at the crack tip if the path is chosen around the outer bounds of the tested specimen, allowing the determination of *J* at the crack tip from the outer loading conditions. Furthermore, the *J*-integral and the ERR are equivalent if the crack grows straight ahead, the deformation at the crack tip is largely linear elastic, and only small-scale yielding is present [18].

In the following, we will briefly present the necessary equations for the evaluation of the conducted experiments in modes I and III and state the most important findings of prior studies. For more detailed derivations, we would like to refer to relevant literature, e.g., [10,11,14,19,20].

**Figure 2:**
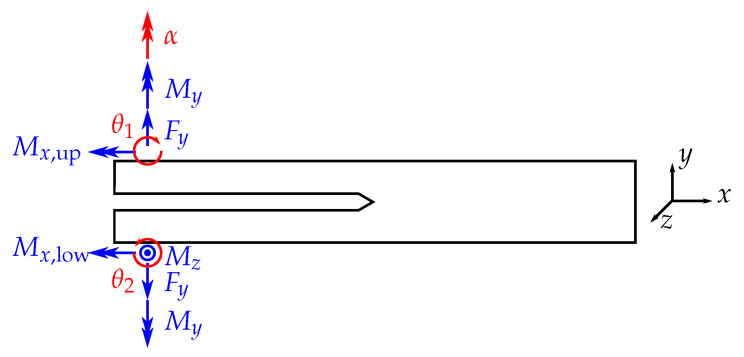
Schematic drawing of a loaded specimen. Measured forces and moments are displayed in blue color; measured or prescribed rotations are displayed in red.

Consider a specimen with the corresponding external loads as displayed in Figure 2. Under these given forces *F* and moments *M*, the *J*-integral yields

(3) $$J=\frac{F_{y}\left(\theta_{1}+\theta_{2}\right)}{b}+\frac{M_{y}\kappa }{b}+\frac{M_{x,\mathrm{up}}^{2}+M_{x,\mathrm{low}}^{2}}{2b}\frac{1}{\mu I_{t}}+\frac{M_{z}^{2}}{2b}\frac{1}{EI_{z}}$$

with the measured rotational angle at the points of load introduction $\theta_{i}$ and the curvature κ. *E* and μ denote the elastic Young’s and shear modulus of the specimen. $I_{z}$ and $I_{t}$ are the second moment of area around the *z*-axis and the torsional second moment of area around the *x*-axis, respectively. By deconstructing the above equation, one obtains the contributions to *J* in the individual modes:

(4) $$J_{I}=\frac{F_{y}\left(\theta_{1}+\theta_{2}\right)}{b}$$

is the contribution in pure mode I,

(5) $$J_{III}=\frac{M_{y}\kappa }{b}$$

is the pure mode III contribution,

(6) $$J_{I^{*}}=\frac{M_{x,\mathrm{up}}^{2}+M_{x,\mathrm{low}}^{2}}{2b}\frac{1}{\mu I_{t}}$$

is an unintended contribution to *J* by an “out-of-plane mode I”—loading due to the specimen twisting, and

(7) $$J_{I+II}=\frac{M_{z}^{2}}{2b}\frac{1}{EI_{z}}$$

is an unintended contribution in modes I and II due to the finite width of the specimen.

The instantaneous experimental determination of the ERR based on the *J*-integral is relatively unsophisticated in mode I, as only force and rotational angle at the points of load introduction have to be measured [10]. To obtain the curvature κ in mode III, an increased experimental effort and the use of additional measuring equipment are required. Provided that the load is introduced to the crack tip by linear-elastically deforming beams, this experimental effort can be reduced by using the Irwin–Kies Equation

(8) $$G_{III}=\frac{M_{y}^{2}}{2b}\frac{dC_{III}}{da}$$

with the change of compliance $dC_{III}$ in shear direction, and the change of the lever arm da during crack propagation [14]. Loh and Marzi were able to establish a connection between the two evaluation methods from Equations (5) and (8) using Bernoulli beam theory, and determined that $J_{III}=G_{III}$ for this evaluation method [14]. Their beam theory approach yields

(9) $$\kappa =\frac{M_{y}}{2}\frac{dC_{III}}{da},$$

offering the possibility to significantly reduce the experimental effort, if $dC_{III}/da$ is determined beforehand.

Furthermore, it should be mentioned that the unintended contributions due to the mode III load were found to be negligible in the studies of Loh and Marzi [14,20,21], in which adhesives were investigated. Because of the changed specimen geometry we use within our study, it must be investigated to which extent these unintended contributions are present at the point of material failure with our proposed mode III setup to determine the “purity” of the mode III fracture.

## 3. Materials and Methods

### 3.1. Tested Material—Polyoxymethylene Homopolymer

The investigated material is a POM-H (Delrin 111PF) from DuPont (DuPont de Nemours, Wilmington, NC, USA) supplied as tubular granules, with a nominal diameter of 4 mm and a nominal height of 2 mm. POM-H shows good mechanical properties (Young’s Modulus *E* around 3500 MPa and Poisson’s ratio ν around 0.42) [1,22,23,24], high ductility down to 0 ${}^{{}^{\circ}}$C, high abrasion resistance and a low friction coefficient. Furthermore, the material is able to resist high amounts of constant loading or fatigue loading. Therefore, POM-H is used in more advanced applications with higher requirements concerning the material properties. POM-H is categorized as a technical thermoplastic material. In preliminary investigations, a glass transition temperature for the tested POM-H of −64 ${}^{{}^{\circ}}$C was obtained using dynamic mechanical thermal analysis.

### 3.2. Specimen Preparation

**Figure 3:**
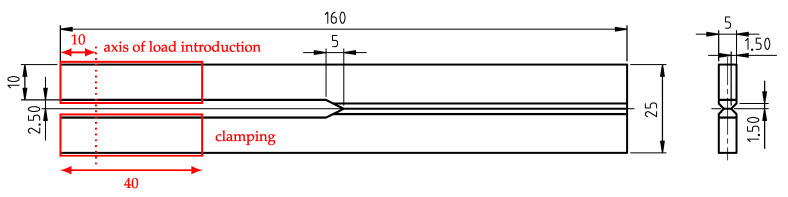
Geometry of the tested polyoxymethylene homopolymer (POM-H) specimens (in mm). The specimens were notched via pushing with a thin razor blade (notch depth ≈ 1 mm).

The geometry of the tested POM-H specimens is displayed in Figure 3. The specimens were milled and cut from plates (dimensions approx. 200 × 200 × 5.5 mm), which were produced by compression molding (Hydrostat 300, Schwabenthan, Germany) with an immersion edge tool (TT-260, Tool-Temp). We want to note that extreme caution was taken during the milling and cutting processes, to ensure minimum temperature yield. For a better overview, the different stages of processing as well as the used tools and milling parameters are disclosed in Appendix A.

Prior to experimental investigation, the specimens were notched via pushing with a thin razor blade. The depth of the notches lay in the range between 1.0 ± 0.4 mm (mean and standard deviation). We want to mention that notching proved to be very difficult because of the brittle material behavior, leading to a larger standard deviation of the achieved notch length.

**Figure 4:**
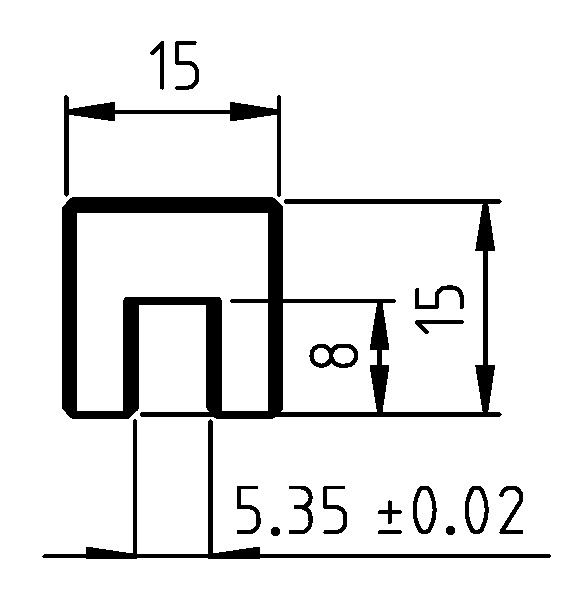
Geometry of the aluminum specimen holder (in mm). The nominal geometrical moments of inertia for the given geometry equate to $I_{y}=4116.66$ mm^4^, $I_{z}=3343.02$ mm^4^, and $I_{t}=7459.68$ mm^4^ around the area’s centroid.

In the mode III investigation, aluminum reinforcements with a length of 160 mm and the cross-section area displayed in Figure 4 are connected to the POM-H specimens. The aluminum reinforcements were milled from an aluminum alloy (AlZn5.5MgCu, material grade number 3.4365) by a professional supplier (Feiler GmbH, Ehringshausen, Germany). The aluminum reinforcements were added to avoid energy dissipation outside of the crack tip due to plastic deformations in the lever arms caused by the introduction of an external moment. Furthermore, as the aluminum reinforcements are very much stiffer than the POM-H specimens and behave linear-elastically, it is ensured that possible influences of material nonlinearities are repressed so that the evaluation of the mode III ERR remains possible with Equation (8).

**Figure 5:**
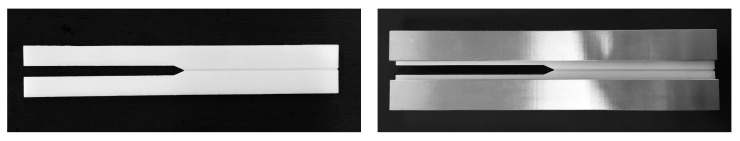
Specimen in mode I investigation (**left**) and mode III investigation (**right**). In mode I, the specimen is clamped directly into the test setup whereas in mode III, aluminum specimen holders are added.

The POM-H specimens and the reinforcements were joined using thermal clamping. For this purpose, the specimens were cooled to a temperature of −60 ${}^{{}^{\circ}}$C with an ultra-deep freezer Herafreeze Basic (Thermo Electron LED GmbH, Langenselbold, Germany) and, hence, subjected to thermal shrinkage. After two to three hours of cooling, the POM-H specimens were removed from the industrial freezer and inserted into the aluminum reinforcements. Then, steel gauge tape with a thickness of 0.20 mm is inserted into the small gaps between specimen and reinforcement on each side. Through thermal expansion of the POM-H specimens, the specimens and the aluminum reinforcements are, hence, uptight at room temperature. It should be noted that the glass transition temperature of POM-H is close to being reached during the cooling procedure, which could, although deemed very unlikely, influence the fracture behavior observed in the experiments. For reasons of space, the investigation of this influencing factor is postponed to possible future works. For a better overview, the specimens in the mode I and mode III investigation are shown in Figure 5.

Overall, 29 specimens were produced (eleven for the mode I investigation, 18 for the mode III investigation).

### 3.3. Experimental Setups and Test Evaluation

**Figure 6:**
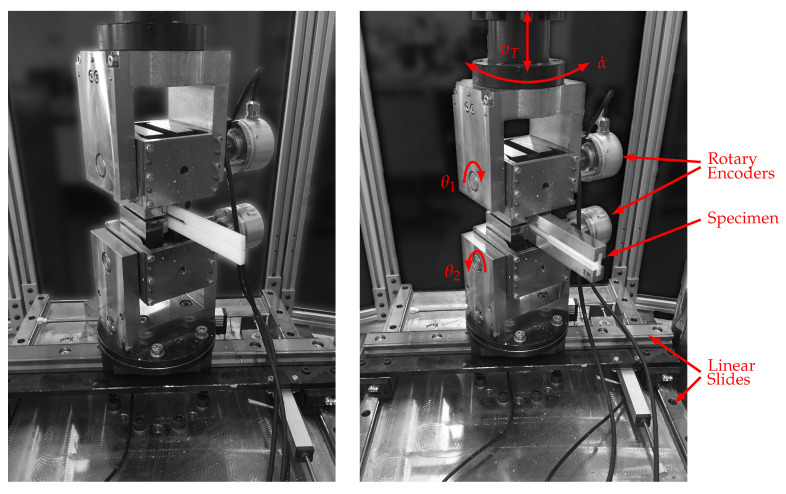
Experimental setup with POM-H specimens. Mode I setup (**left**) and mode III setup (**right**).

In Figure 6, the test setup as realized in a biaxial tension-torsional servo-hydraulic test machine of type MTS Landmark Bionix (MTS Systems, Eden Prairie, MN, USA) is displayed. For both the mode I and the mode III setup, a distance between the point of load introduction and crack tip of 70 mm was maintained. It should be noted that for every tested specimen, the width of the specimen at the initial crack tip was measured with an optical microscope prior to evaluation. All tests were performed under laboratory conditions (20–23 ${}^{{}^{\circ}}$C, approx. 40% RH).

For the mode I tests, the specimen is clamped directly into the test machine without attaching reinforcements. The angles at the points of load introduction $\theta_{1}$ and $\theta_{2}$ are measured with two high resolution rotary incremental shaft encoders. A cross head velocity of $v_{T}=0.05$ mm/s was selected during the mode I investigation. To determine the critical ERR in mode I, eleven specimens were tested. The tests were terminated after the first brittle crack extension for being able to measure the generated crack length after the experiment.

Next to the determination of the mode I ERR using the *J*-integral, the modified beam theory approach according to ASTM standard D5528 [25] is also used to determine the ERR. In this method of data reduction, the ERR is calculated from

(10) $$G_{I}=\frac{3}{2}\;\frac{F\delta }{b(a+\Delta )}$$

with the correction term Δ, which is derived from a regression between the cube root of compliance and crack length. Prior to the analysis, the displacement measurement was corrected with the experimentally obtained compliance of the test setup (561.6 Nmm).

In mode III, the specimen is loaded with an angle-rate of $\dot{\alpha }=0.05$ deg/s. To achieve pure mode III and avoid lateral forces, the setup contains two orthogonal linear slides below the bottom clamping device. Furthermore, axial forces are controlled to be naught by the control system of the test machine, leading to a vanishing mode-I-contribution. To directly obtain the mode III ERR from the *J*-integral, the curvature of three specimens is measured using two strain-gauges in half-bridge circuit at the external surface of the aluminum reinforcements. With the measured averaged strain ε and the width *c* of the aluminum parts, the curvature is computed with κ=2ε/c. Thereupon, $dC_{III}/da$ is determined from the given measured values in order to enable the calculation of the ERR via Equation (8), which leads to a reduction of experimental requirements, as the external moment is solely needed for evaluation. At last, using 15 more specimens, the critical mode III ERR of POM-H is determined.

### 3.4. Crack Length Determination and Fracture Surface Analysis

The produced crack length during the experiment in mode I was determined after testing. Therefore, the crack path of the mode I specimens, which were not fully fractured, were covered with a black ink to mark the crack advancement. Afterwards, the specimens were completely broken and the crack length was obtained as the length of the covered area via a digital calliper (Kellner & Kunz AG, Wels, Austria), with a measurement accuracy of 0.02 mm.

To obtain more information about the fracture process, a detailed analysis of the fracture surfaces was conducted. Therefore, an optical microscope SZX12 (Olympus Life Science Europe GmbH, Hamburg, Germany) was used. The investigation of the fracture surface provides valuable information about the fracture mechanisms and crack path formations in modes I and III.

## 4. Results and Discussion

### 4.1. Experimental Results in Mode I

**Figure 7:**
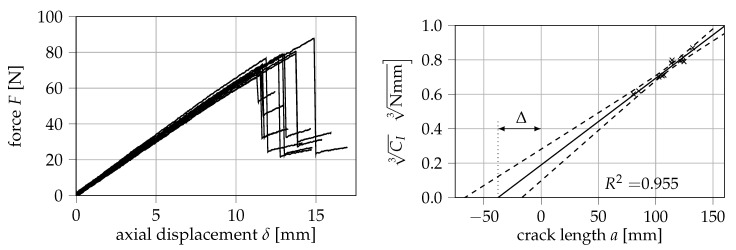
Measured force-displacement curves in mode I (**left**) and cube root of compliance over crack length with 5% confidence bands (**right**). Eleven specimens were tested.

In Figure 7, the measured force-displacement curves are displayed. As shown, the relationship between force and displacement is almost completely linear up until the point of a sudden, brittle fracture. Furthermore, the cube root of the specimen compliance is displayed over the crack length with the 5% confidence bands of the performed regression, from which a correction factor Δ of 37.66 mm can be derived. Using the modified beam theory approach according to Equation (10) yields a critical ERR of (6.58 ± 1.09) kJ/m^2^.

**Figure 8:**
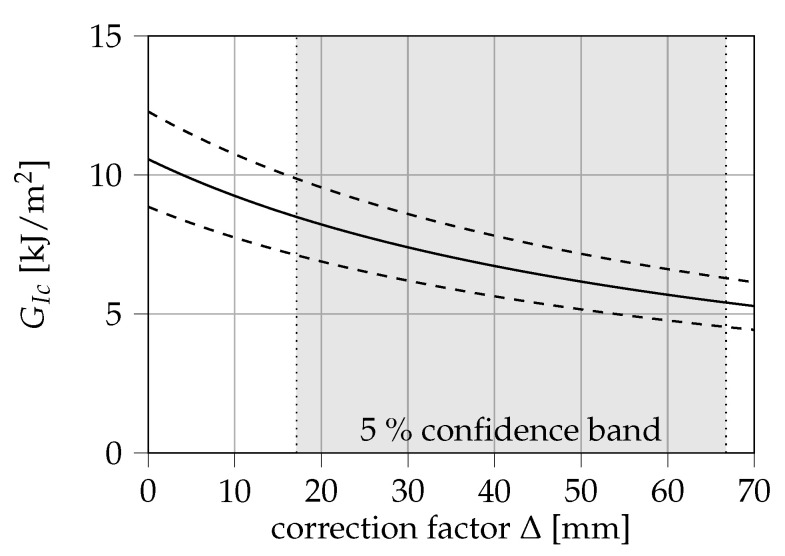
Influence of the correction factor Δ on the energy release rate (ERR) obtained from modified beam theory.

However, the confidence bands suggest that the determination of Δ leads to a considerably large uncertainty in the calculation of $G_{Ic}$ in this case. To roughly quantify this uncertainty, the critical ERR and its standard deviation depending on the obtained correction factor Δ is displayed in Figure 8. At the 5% confidence bands of Δ, ERRs between (5.18 ± 0.85) kJ/m^2^ and (8.13 ± 1.34) kJ/m^2^ are obtained. It is therefore argued that the calibration of the corrected crack length in this method of data reduction may be a strong source of error, which would be eliminated in the calculation of the *J*-integral via Equation (4).

**Figure 9:**
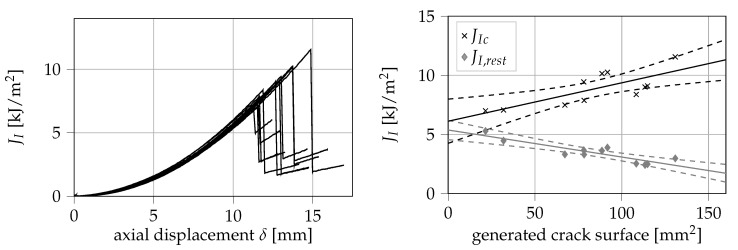
Measured values of the mode I *J*-integral (**left**) and obtained values for $J_{Ic}$ and $J_{I,\mathrm{rest}}$ over the generated crack surface (**right**).

The measured mode I *J*-integral is shown over the cross head displacement δ in Figure 9. From the conducted experiments, a value for $J_{Ic}$ of (8.84 ± 1.39) kJ/m^2^ can be obtained. On the right side of Figure 9, both the maximum values of $J_{I}$ as well as the values at crack arrest $J_{I,\mathrm{rest}}$ are displayed over the generated crack surface. Interestingly, these results imply that $J_{Ic}$ increases linearly with the generated crack surface, whereas $J_{I,\mathrm{rest}}$ linearly decreases. In Table 1, the parameters of both of these linear regressions are summarized. Both regression lines just narrowly fail to meet in the intersection with the ordinate axis. However, within the confidence bands of the regression analyses, it is possible that an intersection of both lines exists on the ordinate axis. One may hypothesize that this intersection is the minimum possible value of *J* able to cause crack propagation. Considering the overlaps between both confidence bands, the intersection is most likely located in the range between 4.56 and 6.19 kJ/m^2^.

In practical application, these results can be advantageous: Firstly, knowing the relationships between $J_{Ic}$ and $J_{I,\mathrm{rest}}$ with the generated crack surface allows a rough determination of the fracture energy of a thin-walled component (with a similar geometry to the tested specimens) in hindsight, by simply measuring the area of the fracture surface. Secondly, the intersection between the regression lines is a useful parameter for the design of a thin-walled polymeric component, as it represents a lower limit to the crack driving force. We suggest that further research is performed in these areas to determine whether the obtained results also remain valid for larger sample sizes, other specimen geometries, and different polymeric materials.

### 4.2. Experimental Results in Mode III

#### 4.2.1. Determination of $dC_{III}/da$

**Figure 10:**
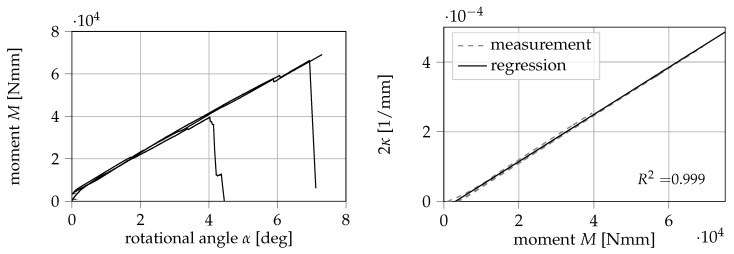
Measured moment $M_{y}$ over the rotational angle α (**left**) and linear regression between the curvature 2κ and the measured moment $M_{y}$ (**right**) obtained with three specimens.

Within this study, three specimens are used to experimentally determine the constant $dC_{III}/da$. In Figure 10, the measured moment *M* is displayed over the rotational angle of the biaxial testing machine α.

As can be seen in Equation (9), $dC_{III}/da$ can be obtained from the relationship $2\kappa /M_{y}$. Here, $dC_{III}/da$ is determined from a linear regression between 2κ and $M_{y}$ up until the measured maximum of $J_{III}$. The value for $dC_{III}/da$ is then derived from the slope of the regression line. The measured values of curvature κ on the outside of the reinforcements and the bending moment $M_{y}$ as well as the performed regression are also displayed in Figure 10. The slope of the regression line equates to (6.75 ± 0.01) × 10^−9^ 1/Nmm^2^ (±5% confidence interval of regression slope). It can be observed that the measured values are captured by the regression line with great accuracy (correlation coefficient $R^{2}=0.999$).

For verification purposes, $dC_{III}/da$ is also determined analytically with $dC_{III}/da=2/\left(EI_{y}\right)$, the aluminum’s elastic modulus of E=(70±1) GPa and the specimen holders area moment of inertia $I_{y}=4116.66$ mm^4^. From this, a value of $\left(6.940\pm 0.101\right)\times 10^{-9}$ 1/Nmm^2^ can be derived, which fits with the experimentally obtained $dC_{III}/da$, given that the compliance of the aluminum reinforcements itself should be greater than the compliance of the holder with an inserted POM specimen. Because of the relatively good agreement between experimentally measured and analytically determined $dC_{III}/da$, the evaluation of the unintended contributions according to Equations (6) and (7) is performed with the moments of inertia of the reinforcements.

#### 4.2.2. Critical Mode III ERR

**Figure 11:**
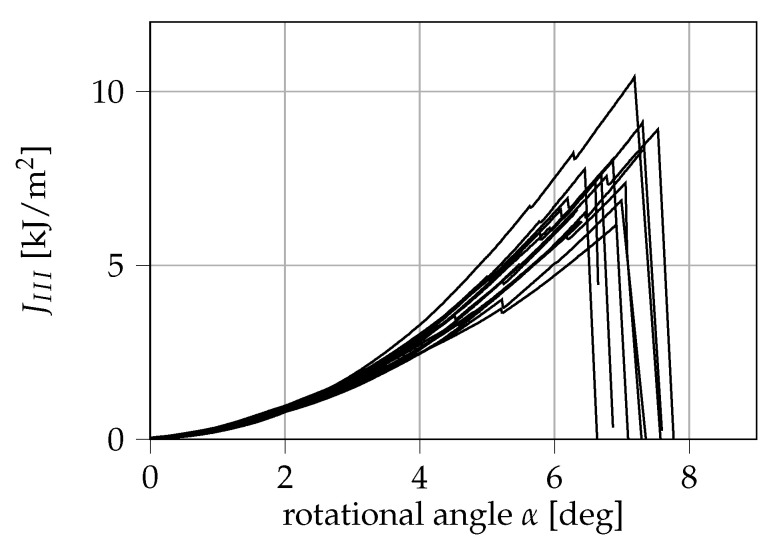
Experimental results of the mode III investigation. The shown mode III ERR of 15 specimens was determined from the Irwin-Kies Equation.

The experimental results with 15 additional specimens are displayed in Figure 11. From this, a critical mode III ERR of (7.59 ± 1.19) kJ/m^2^ can be derived. We note from the diagram that prior to critical failure all samples show at least one significant, sudden drop in $J_{III}$. This so-called pop-in phenomenon has been mostly observed for steels or weldments in mode I testing. Studies found that pop-ins can both result from a local unstable crack growth that is then stabilized by the surrounding material [26] or by the formation of cracks perpendicular to the plane of the initial pre-crack [27]. To our knowledge, pop-in in pure mode III fracture of polymers is observed for the very first time within this study.

**Figure 12:**
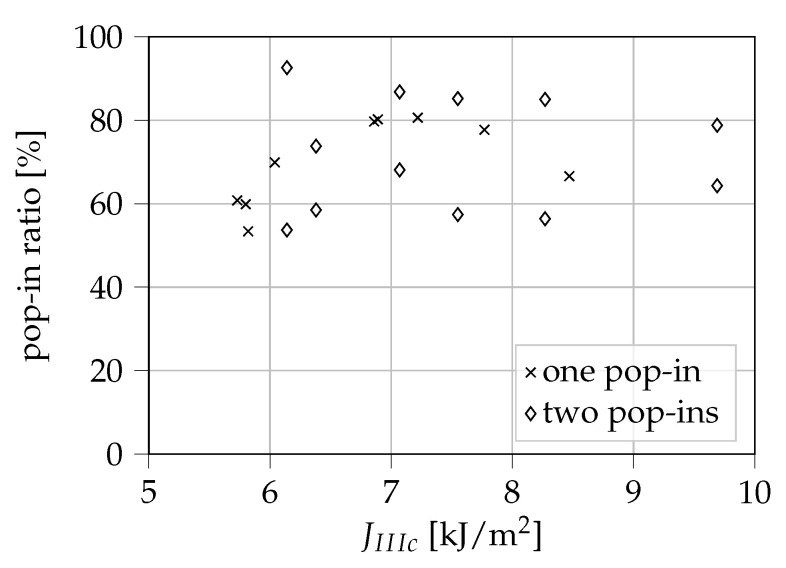
Ratio between the observed pop-in values of $J_{III}$ and $J_{IIIc}$.

In Figure 12 the ratio between the values of $J_{III}$ at which pop-ins occurred and $J_{IIIc}$ is displayed. Here, pop-in ratios between 53% and 93% can be observed. For the given sample size, the results do not allow any statement as to whether the pop-in-ratio is dependent on the measured $J_{IIIc}$. The occurrence of pop-ins can be critical for the structural integrity of a thin-walled POM-H component loaded in shear as cracks could grow prior to critical failure, potentially weakening thin-walled components to a significant extent. Because pop-ins were already observed shortly above 50% of $J_{IIIc}$, it is indicated that shear loads could be very much critical for a thin-walled POM-H component.

**Figure 13:**
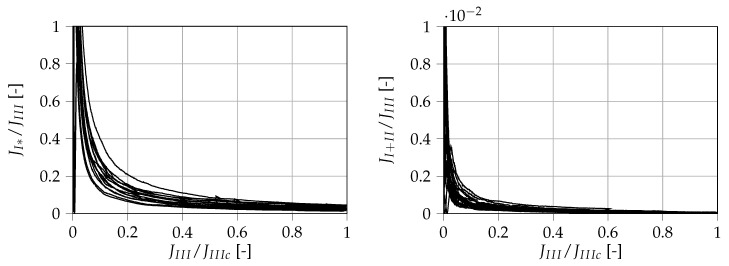
Artificial contributions determined within the mode III investigation. The unintended out-of-plane mode I load $J_{I^{*}}$ (**left**) and the mode I/II contribution $J_{I+II}$ (**right**) are displayed normalized on $J_{IIIc}$ over $J_{III}/J_{IIIc}$.

The measured unintended contributions normalized to the current measured mode III ERR are displayed over $J_{III}/J_{IIIc}$ in Figure 13. One should note that the contribution of $J_{I^{*}}$ is considerably larger than the measured mode III ERR at the start of the test. This may be partly attributed to the fact that the breakaway force of the linear slides must first be overcome at the beginning of the test. The load history is therefore not to be considered as a pure mode III loading process. However, it can also be observed that the unintended contributions are, in fact, negligible at the points of fracture. At fracture, $J_{I^{*}}$ takes up (3.02 ± 1.01) % of $J_{III}$ and tends further towards naught, whereas $J_{I+II}$ only takes up (0.04 ± 0.04)‰. This means that, according to the measurements, the fracture process can be considered as a pure mode III fracture.

### 4.3. Approximate Determination of the Stress Intensity Factors $K_{Ic}$ and $K_{IIIc}$

As a side effect, for the special case of linear elastic, isotropic, and brittle or quasi-brittle materials, the stress intensity factors in mode I and mode III can directly be related to the critical values of *G* (and *J*) in a given loading mode. In mode I, the stress intensity factor $K_{Ic}$ is calculated with

(11) $$\begin{array}{c} K_{Ic}=\sqrt{E^{\prime }J_{Ic}}=\sqrt{E^{\prime }G_{Ic}} \end{array}$$

with $E^{\prime }=E$ in plane stress and $E^{\prime }=E/\left(1-\nu^{2}\right)$ in plane strain. Unfortunately, it is unclear at this point, whether the crack tip was loaded in plane stress or plane strain due to the necessary addition of the side-grooves to the specimen. Although the thin geometry of the specimens should induce a plane stress state, the addition of side-grooves is known to induce stress triaxialities that could lead to a state of plain strain [28].

In mode III, i.e., for antiplane shear, the stress intensity factor $K_{IIIc}$ can be derived with

(12) $$\begin{array}{c} K_{IIIc}=\sqrt{2\mu J_{IIIc}}=\sqrt{2\mu G_{IIIc}}. \end{array}$$

As shown in Section 4.1, a critical ERR $J_{Ic}$ of (8.84 ± 1.39) kJ/m^2^ was determined from the experiments performed in this study, which roughly equates to a mode I stress intensity factor $K_{1c}$ of 5.6 MPa m^1/2^ (plane stress) or $K_{Ic}$ of 6.1 MPa m^1/2^ (plane strain), respectively. In mode III, a fracture energy of (7.59 ± 1.19) kJ/m^2^ was obtained, which equates to a $K_{IIIc}$ of about 4.3 MPa m^1/2^.

### 4.4. Summary and Discussion of the Obtained Fracture Mechanical Properties

In this section, we want to shortly summarize and discuss the obtained fracture mechanical properties displayed in Table 2.

In the mode I investigation, a relatively large discrepancy between the experimentally obtained mode I $G_{Ic}$ and $J_{Ic}$ was found. The authors believe that this discrepancy is due to a large uncertainty in the determination of the corrected crack length Δ from the crack length measurement and specimen compliance. We want to point out that using the *J*-integral method of evaluation offers the possibility to determine the fracture energy of a polymeric bulk specimen without measuring crack length or the elastic properties of the material. As only force and rotational angle have to be measured for the determination of the mode I *J*-integral, a simultaneous determination of the fracture energy during the experiment is possible. This allows controlling the experiments on specific values of *J*, which poses an interesting topic for future research.

Regarding the obtained mode III *J*-integral, we want to point out two important factors: Firstly, we want to emphasize that the evaluation of the *J*-integral with the measured curvature κ might be necessary in some cases. With the aluminum reinforcements, a constant $dC_{III}/da$ could be ensured in this study, rendering $J_{III}$ and $G_{III}$ equivalent. However, this may change if the reinforcements are removed. Furthermore, if the material was more ductile, a determination of $J_{III}$ with the curvature κ may prove to be more accurate. Secondly, it should also be mentioned that $J_{III}$ can be determined with both evaluation methods (κ or $dC_{III}/da$) instantaneously during the experiment. This, as in mode I testing, allows controlling the experiments on specific values of *J* in future studies.

A review of literature provided mode I stress intensity factors $K_{Ic}$ for POM-H between 2.5 and 6.9 MPa m^1/2^ [4,6,7]. Thus, the obtained mode I stress intensity factor between 5.6 and 6.1 MPa m^1/2^ lies within the range of reference measurements from literature. We want to emphasize that our results match with the plane strain fracture toughness of the same material determined with compact tension specimens [6,7]. Unfortunately, we were unable to better investigate the stress state at the crack tip within the scope of this work, and we cannot report the mode I stress intensity factor at a greater accuracy. To the authors’ knowledge, no comparative values are available under pure mode III. Hence, one of the highlights of our study is that the determination of the pure mode III fracture toughness of POM was made possible with our setup for the first time. However, future studies on the topic are suggested in order to verify the determined mode III fracture energy and fracture toughness.

### 4.5. Investigation of the Fracture Surfaces

#### 4.5.1. Mode I

**Figure 14:**
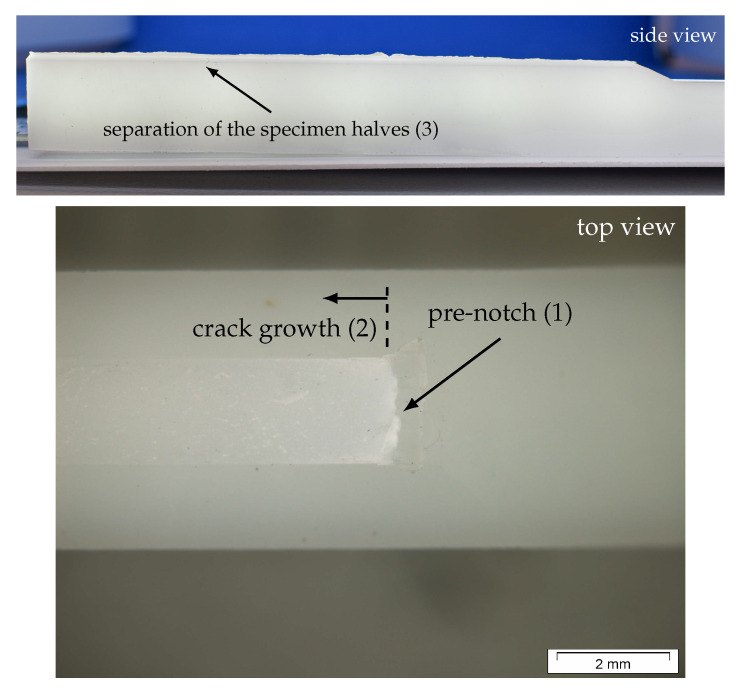
Fracture surface images from a monotonically loaded mode I specimen analyzed using a light microscope (16× magnification)—three areas of different crack areas were observed: pre-notch before testing (1), the crack growth area (2) and the area produced during the separation of the two specimen halves (3).

An optical analysis of the fracture surface was conducted in this study to gain more information about ongoing crack growth mechanisms during testing. This is a common method to investigate the crack growth process after testing and to identify changes in the latter with the surface structure. An overview picture of the fracture of a specimen tested in mode I and a light microscope image of the fracture surface close to the initial notch are shown in Figure 14.

The macroscopically observed fracture surface of monotonically loaded mode I specimens is flat (see overview picture in Figure 14), with three different areas on the fracture surface (marked with (1) to (3) on the images in Figure 14). The first area represents the pre-notch, which was generated via a razor blade before testing. Area (2) marks the produced crack growth during testing and area (3) was generated after testing to determine the ligament length.

The quality of the pre-notch (sharp crack tip without any signs of deformation) has a major influence on the resulting fracture mechanical parameters. Therefore, it is of high interest to guarantee a similar notch quality for all investigated specimens. For the used POM-H specimens, the notching procedure was found to be challenging because of the rather brittle material behavior. This difficulty manifests itself as small imperfections at the crack tip, as shown in Figure 14. Hence, small variations of the produced pre-notch led to an increased standard deviation of the determined fracture mechanical parameters.

No indications of plastic deformation, which would have been visible as intensive white zones on the fracture surfaces, were found in the investigation. Furthermore, as already observed during the experimental investigation, the specimens fractured in a brittle manner. This observation is in contrast to previous monotonic mode I tests on the same material class [6], in which an intensive white zone was found whose formation is attributed to crazes and micro-voids within the material.

In general, deformation rate, specimen thickness, and the added side-grooves also have a large influence on the size of the plastic zone. Based on the fracture surface investigation and the brittle fracture without any indications of plastic deformations, it is suggested that the specimens fractured in a plane strain state. This is also supported by the addition of the side-grooves, which lead to an increased triaxiality along the crack front. However, this finding suggests that further investigations are required to gain a better understanding of the influence of specimen thickness and groove shape on the fracture behavior of POM-H.

#### 4.5.2. Mode III

**Figure 15:**
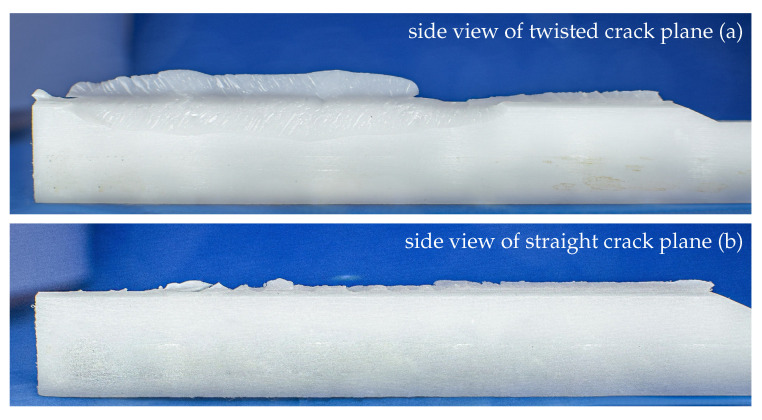
Fracture surface images from monotonically loaded mode III specimens (side view)—two different types of surface structure were observed: twisted crack planes along the fracture surface (**a**) and a nearly straight crack plane with little deflections (**b**).

To examine the fracture process of the mode III samples, a fracture surface analysis via an optical microscope was conducted similar to the mode I procedure. An overview of two representative fracture surfaces loaded in mode III is shown in Figure 15. Interestingly, two different types of mode III fracture surfaces were observed. A larger group of the mode III specimens displayed extensive twisting and crack plane deflection (Figure 15a), whereas in some cases, a less deflected crack flank was observed (Figure 15b).

The twisting of the crack during propagation is known as mode I branching. Here, the crack deflects under mixed-mode conditions to a local mode I loading [29,30]. Such mode I branches were also observed in a previous study on the same material in mode I/III fatigue tests [8]. Furthermore, the occurrence of shear lips and slant crack growth is quite common for thin specimens subjected to mode III [31,32,33]. As the measurements obtained in Section 4.2.2 demonstrated that the fracture was initiated in pure mode III and the fracture surfaces show typical signs of mode III fracture and the subsequent mixed-mode crack propagation, we can conclude that our adaption to the ODCB test allows the determination of the mode III fracture energy without further restrictions.

After a comparison with the results shown in Figure 12, a dependency of the macroscopically observed angle of the fracture surface and the pop-in ratio is suggested. A small ratio of $J_{III,\mathrm{pop}-\mathrm{in}}/J_{IIIc}$ seems to lead to a more flat and straight fracture, as shown in Figure 15b. This means that the macroscopically twisted and deflected fracture surfaces may be related to a higher pop-in ratio. However, to prove this assumption, further investigations are necessary. Especially a determination of the twisting angle at the crack tip using optical methods could be of huge benefit. Apart from the implied change in pop-in ratio, no connection between other experimental results and the twisting angle of the fracture surfaces could be found within the framework of this study.

**Figure 16:**
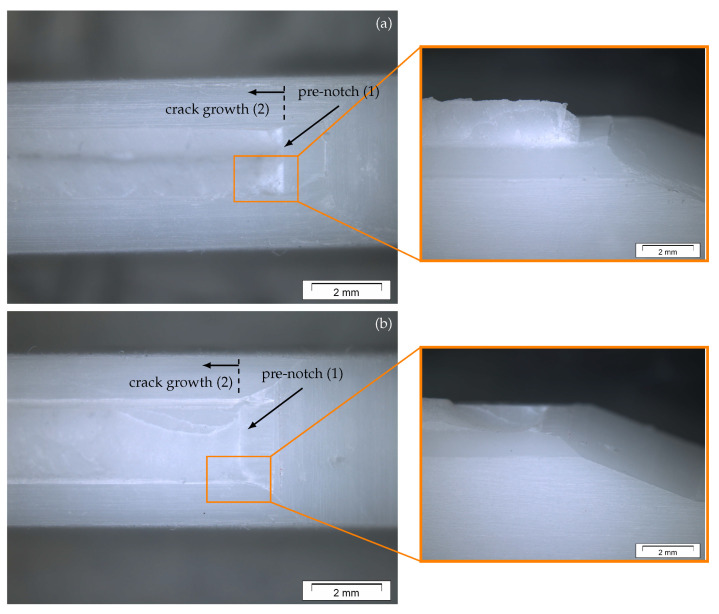
Fracture surface images from two mode III specimens analyzed using a light microscope (16× magnification). Image of a deflected fracture surface (**a**) and a flat fracture surface (**b**)—two different crack areas were observed on the fractured specimen half: pre-notch before testing (1) and area of crack growth during testing (2).

Akin to the mode I fracture, no indications of plastic deformation were observed on the mode III fracture surfaces. Figure 16 presents a detailed picture of the fracture surfaces of a macroscopically twisted specimen (Figure 16a) and a specimen with a less deflected fracture surface (Figure 16b). The pre-notch is marked as area (1) and the mode III crack growth is marked as area (2).

### 4.6. Advantages, Limitations and Research Proposals

Before summarizing the most important results obtained within our paper, we would like to discuss the advantages and limitations of our work and propose topics for future studies.

We are aware that our research may have limitations: As we mainly tried to enable the testing of thin-walled polymeric components, we were unable to examine some possible influencing factors in more detail, unfortunately. It is generally well known that both the thickness of the specimen as well as the position of the initial crack tip may have a large influence on the fracture behavior. Furthermore, the stress state at the crack tip is likely influenced by the geometry of the grooves on the side of the specimen. As previously mentioned, the absence of signs of plastic deformation at the crack tip suggests a fracture in plane strain, although the slender specimen geometry should lead to a plane stress state. Hence, the impact of the grooves’ geometry is unclear at this point. In this case, a simulative study using finite element analysis should provide important insights, but as we primarily focused on the experimental setup and the methods of evaluation, we have refrained from performing simulations at this point in time. Such detailed analyses would not have been possible within the framework of this study without neglecting other important areas of our investigations. We propose that further research be undertaken in these areas.

In our investigation, we had to rely on thermal clamping of the specimens. Currently, we also cannot certainly rule out that the cooling procedure has influenced the overall fracture behavior of the specimen in mode III. Furthermore, one could argue that the addition of the aluminum reinforcements influences the load introduction to the crack tip, possibly impacting the measured results. We are aware of this limitation and, hence, propose that further research is undertaken to improve the specimen geometry. An alternative can be a change of specimen thickness at the lever arms, which may allow omitting the aluminum reinforcements in the first place.

As discussed above, the instantaneous determination of *J* during the experiment in both modes I and III can be used to control the experiments on *J*. This enables testing in mixed-mode I+III under constant mixed-mode ratios, which can help to better understand the fracture behavior of polymeric bulk specimens. A revision of the sample geometry will therefore be required so that the aluminum holders become obsolete. In future studies, this should be investigated in more detail.

We also want to emphasize that the proposed setups should also be tested with other polymeric materials, as we exclusively focused on POM-H in our research. The selection of POM for this study was mainly due to its high relevance among engineering plastics and our prior knowledge of the material. Another advantage of POM is its limited dependency on strain rate in the elastic range [34], which allows the strain energy density to be considered as rate-independent in good approximation.

One of the obvious advantages of our methodology is that using the *J*-integral allows for the investigation of ductile materials. As a nice side effect, in case of a brittle or quasi-brittle failure, the stress intensity factors in modes I and III can be determined. Furthermore, we found evidence to suggest that our mode III setup enables pure mode III testing of various kinds of polymers, as our setup allows for a precise experimental determination of the contributions to the fracture process in other fracture modes.

## 5. Conclusions

Within the limitations of our study, the following conclusions can be drawn:

- Our proposed methodology provides the possibility to determine the energy release rate of a polymeric material in pure mode I on the basis of the *J*-integral. It also allows us to measure the energy release rate in pure mode III, which is not yet possible with setups found within literature. For isotropic, brittle or quasi-brittle materials, the results can approximately be converted into the stress intensity factors in mode I and mode III.
- The observed pop-in fracture in mode III could be crucial for the structural integrity of thin-walled POM-H components. Crack growth prior to reaching the critical energy release rate can significantly weaken a structure when loaded in shear.
- The analysis of the fracture surfaces showed no signs of plastic deformation close to the initial notch in both modes I and III. The mode I specimens displayed a macroscopically flat fracture surface, whereas the mode III samples showed a deflection of the crack plane. The twisting of the crack path was attributed to a mixed-mode crack propagation and requires further investigation.

Additionally, we suggest that further research should be undertaken in the following areas:

- Future studies should address the applicability of the presented test setups to other materials, especially more ductile polymers.
- A simulative study of the experimental design could provide information on the validity of the test setups proposed in this study. Furthermore, an investigation of the influence of thermal clamping and the stress state at the crack tip using finite element analysis is suggested.
- The influences of specimen thickness, initial crack position, groove geometry, and influences due to notching have not been investigated within this study. We suggest that further studies focus on this, as their influence on the fracture behavior determined from the given test setup is not yet foreseeable.
- The test setups can be modified and “superimposed” to achieve a mixed mode I/III load. For this, it may be necessary to revise the clamping procedure.
- The fracture surface investigation should be expanded to obtain more information about the influence of pop-ins and the deflection of the crack-plane in the mode III tests.

## Figures and Tables

**Table 1:** Function parameters and correlation coefficients of the linear regressions between $J_{Ic}$ and $J_{I,\mathrm{test}}$ vs. crack length.

	Slope	Intercept	$R^{2}$
	[(kJ/m^2^)/mm^2^]	[kJ/m^2^]	[-]
$J_{Ic}$	3.249 × 10^−2^	6.12	0.583
$J_{I,\mathrm{rest}}$	−2.286 × 10^−2^	5.37	0.783

**Table 2:** Summary of the most important experimentally obtained parameters.

	Mode I	Mode III
$G_{c}$ from modified beam theory	(6.58 ± 1.09) kJ/m^2^	n.a.
critical value of *J*	(8.84 ± 1.39) kJ/m^2^	(7.59 ± 1.19) kJ/m^2^
approx. fracture toughness *K*	5.6 MPa m^1/2^ (plane stress)	4.3 MPa m^1/2^
	6.1 MPa m^1/2^ (plane strain)	
min. of *J* to cause crack propagation	4.56–6.19 kJ/m^2^	n.a.
pop-in ratio	not observed	53–93%

## Abbreviations

The following abbreviations are used in this manuscript:

POM	Polyoxymethylene
DCB	Double cantilever beam
ODCB	Out-of-plane-loaded double cantilever beam
ERR	Energy release rate

## Appendix A. Production of the POM-H Specimens

For a better overview, we want to disclose the manufacturing steps and parameters for the production of the POM-H specimens in greater detail:

- Step 1: Compression molding tubular granules into plates of approx. 200 × 200 × 5.5 mm; Hydrostat 300 (Schwabenthan, Germany) with an immersion edge tool (TT-260, Tool-Temp)
- Step 2: Cutting the pressed plates into a rectangular shape of size 160 × 25 mm; Bäuerle tablesaw KSW-7 (Riston Werkzeug GmbH, Fellbach, Germany)
- Step 3: Milling the rectangles to correct thickness; universal milling machine Deckel FFP3 (Friedrich Deckel AG, München, Germany) with HSS face-milling cutter ø60 mm at 500 rpm
- Step 4: Milling the slit between the lever arms; universal milling machine Deckel FFP3 (Friedrich Deckel AG, München, Germany) with HSS prismatic disk cutter ø250 mm at 80 rpm
- Step 5: Cutting the sidegrooves; Bäuerle tablesaw KSW-7 (Riston Werkzeug GmbH, Fellbach, Germany) with prismatic sawblade
